# A Web-Based Patient Portal for Mental Health Care: Benefits Evaluation

**DOI:** 10.2196/jmir.6483

**Published:** 2016-11-16

**Authors:** Sarah Kipping, Melanie I Stuckey, Alexandra Hernandez, Tan Nguyen, Sanaz Riahi

**Affiliations:** ^1^ Ontario Shores Centre for Mental Health Sciences Whitby, ON Canada; ^2^ Department of Psychiatry University of Toronto Toronto, ON Canada

**Keywords:** efficiency, organizational, electronic health records, mental health, mental disorders, patient activation

## Abstract

**Background:**

Treatment for mental illness has shifted from focusing purely on treatment of symptoms to focusing on personal recovery. Patient activation is an important component of the recovery journey. Patient portals have shown promise to increase activation in primary and acute care settings, but the benefits to tertiary level mental health care remain unknown.

**Objective:**

To conduct a benefits evaluation of a Web-based portal for patients undergoing treatment for serious or persistent mental illness in order to examine the effects on (1) patient activation, (2) recovery, (3) productivity, and (4) administrative efficiencies.

**Methods:**

All registered inpatients and outpatients at a tertiary level mental health care facility were offered the opportunity to enroll and utilize the patient portal. Those who chose to use the portal and those who did not were designated as “users” and “nonusers,” respectively. All patients received usual treatment. Users had Web-based access to view parts of their electronic medical record, view upcoming appointments, and communicate with their health care provider. Users could attend portal training or support sessions led by either the engagement coordinator or peer support specialists. A subset of patients who created and utilized their portal account completed 2 Web-based surveys at baseline (just after enrollment; n=91) and at follow-up (6 and 10 months; n=65). The total score of the Mental Health Recovery Measure (MHRM) was a proxy for patient activation and the individual domains measured recovery. The System and Use Survey Tool (SUS) examined the use of functions and general feedback about the portal. Organizational efficiencies were evaluated by examining the odds of portal users and nonusers missing appointments (productivity) or requesting information from health information management (administrative efficiencies) in the year before (2014) and the year after (2015) portal implementation.

**Results:**

A total of 461 patients (44.0% male, n=203) registered for the portal, which was used 4761 times over the 1-year follow-up period. The majority of uses (95.34%, 4539/4761) were for e-views. The overall MHRM score increased from 70.4 (SD 23.6) at baseline to 81.7 (SD 25.1) at combined follow-up (*P*=.01). Of the 8 recovery domains, 7 were increased at follow-up (all *P*<.05). The odds of a portal user attending an appointment were 67% (CI 56%-79%) greater than that of nonusers over the follow-up period. Compared with 2014, over 2015 there was an 86% and 57% decrease in requests for information in users and nonusers, respectively. The SUS revealed that users felt an increased sense of autonomy and found the portal to be user-friendly, helpful, and efficient but felt that more information should be accessible.

**Conclusions:**

The benefits evaluation suggested that access to personal health records via patient portals may improve patient activation, recovery scores, and organizational efficiencies in a tertiary level mental health care facility.

## Introduction

Mental illnesses are one of the highest contributors to the global disease burden, accounting for the greatest proportion of years lived with disability [1]. Over the past few decades, mental health treatments have shifted from being purely symptom focused to adopt a recovery philosophy—that is, supporting patients in their personal journey of self-discovery and regaining control of their path to wellness [2]. In order for patients to set and achieve their personal wellness goals, they must be activated in their care. In people with schizophrenia, patient activation is correlated with recovery attitudes [3]. Patients and carers with increased activation in their care develop the knowledge, skills, and confidence [4] to manage their illness effectively, which may lead to the engagement in self-management behaviors [5]. There are many challenges to activating patients, and strategies for facilitating activation, recovery, overall well-being, and self-management are needed.

Enhancing access to health care information for patients and their carers promotes active partnership between patients and health care providers. Patient portals linked to a hospital’s electronic medical record (EMR) data repository allow patients and/or designated carers to access their personal health information [6], which may facilitate patient activation. Many positive patient outcomes have been reported following implementation of patient portals, including improved adherence to treatment, reduced medical errors and adverse drug reactions, better communication between the patient and provider, perceived improvement in care quality, increased patient engagement, and an increased sense of autonomy [7-9], although these findings are not consistent across studies [8,10]. To date, studies have been conducted in acute or long-term care settings for people with physical illness, and the effects of implementation of patient portals have not been examined in mental health care. Considering that many of the documented improvements align well with recovery philosophy (ie, increased sense of autonomy, patient engagement, and patient-provider communication), implementation of a patient portal in a mental health care facility would have the potential to positively impact both clinical outcomes and recovery.

Implementation of a patient portal in a health care facility must provide benefit not only to the patients but also to the organization. Patient portals have been proven to have some positive impact on organizational efficiencies. One review reported that portal users had a quicker decline in the rate of office visits and a slower increase in the number of telephone contacts compared with the control group [7], while another reported provider time savings from in-person clinic appointment avoidance owing to portal communication [8]. On the other hand, a realist review showed that there was no decrease in health resource utilization [8]. In 5 of the 8 studies included in the review, health resource utilization increased [11], which could be expected with increased access and use of the system. Thus, it remains unclear whether these effects are positive or negative overall. Furthermore, these reports do not pertain to mental health, which has a different care structure, and portal use may have a different organizational impact on a facility serving this population.

The purpose of this study was to conduct a benefits evaluation of a patient portal for patients undergoing treatment for serious or persistent mental illness. The objectives were to examine the effects on (1) patient activation in care, (2) recovery, (3) productivity, and (4) administrative efficiencies.

## Methods

### Study Design and Setting

This observational cohort study—reported according to STROBE (Strengthening the Reporting of Observational Studies in Epidemiology) guidelines [12] and the CONSORT-EHEALTH (Consolidated Standards of Reporting Trials of Electronic and Mobile Health Applications and Online Telehealth) extension [13]—was carried out at a tertiary level mental health care facility (Ontario Shores Centre for Mental Health Sciences, Whitby, Canada), which offers inpatient (16 units, 326 beds) and outpatient services (>60,000 visits per year in 26 clinics) to those with serious and persistent mental illnesses (see Multimedia Appendix 1) [13]. This study was approved by the Research Ethics Board of Ontario Shores Centre for Mental Health Sciences.

### Recruitment

All inpatients and outpatients (or their carers) receiving care from December 2014 to December 2015 were eligible to register for a portal account and were invited to participate. Patients were approached by the engagement coordinator (clinical educator) and provided with information about the portal. Portal users were defined as those who chose to register in the portal between December 2014 and November 2015. Users were also recruited through the health information management department when patients made contact for their health information, although most were enrolled through the engagement coordinator. The design of the recruitment strategy likely resulted in an overrepresentation of participants with higher technological literacy and/or motivation to participate in their own care and less severe illness. Likewise, those with lower computer literacy or motivation to be involved in their care or more severe illness were likely underrepresented. This bias should be recognized as an important limitation to the study. The first steps of implementation, however, were to assess the functionality and the benefits of using the portal; we therefore decided to first implement with the intent of recruiting early adopters. Work is underway to identify and address barriers to portal use in patients who are more resistant to use. Informed consent was waived by the research ethics board for analysis of deidentified data pulled from the organization’s EMR data repository. In a subset of participants completing Web-based surveys, consent to participate was implied by completing the surveys after being invited by detailed email communication from the study coordinator.

### Portal Design

**Figure 1:**
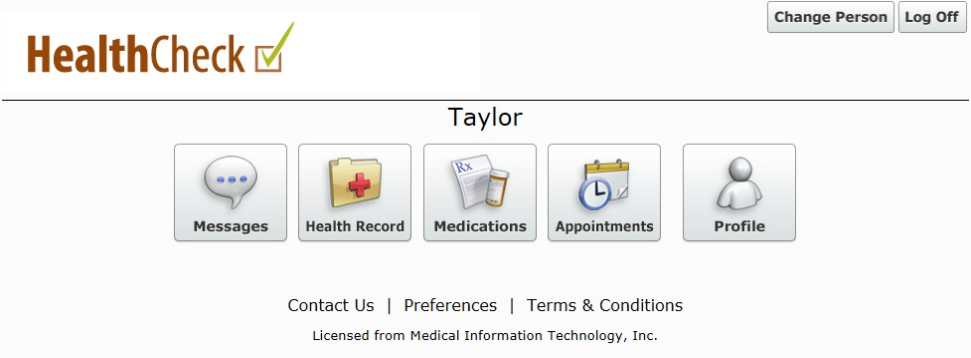
Home page of the patient portal.

The patient portal (Ontario Shores HealthCheck Patient Portal; Figure 1) is a vendor application (Medical Information Technology, Inc (Meditech), Westwood, MA, USA) that accesses personal health information documented in the EMR. It was designed to meet the following objectives: (1) to allow patients access to view their information from the EMR; (2) to give patients another method by which to request medication renewals; (3) to provide patients with the ability to view their outpatient appointments generated through Meditech’s “Community Wide Scheduling” module; (4) to allow patients to conveniently update their demographic or contact information; (5) to provide patients with access to educational materials, such as discharge instructions; (6) to provide a medium for communication between patients and physicians and/or interprofessional outpatient clinician team members; and (7) to maintain flexibility to allow for future development and iterations to meet evolving needs of patients and clinicians. The hospital’s privacy officer (Leader, Privacy and Access) was a member of the project implementation team, and the Information and Privacy Commissioner of Ontario’s Privacy by Design model [14] was operationalized to ensure security.

Users accessed the portal through any Web browser on a computer or electronic device with Internet connectivity. Users were able to show, print, and share their record with health care providers at other facilities in support of maintaining continuity of care. The portal functions included were predetermined by those available from the vendor. For the purpose of this evaluation, 3 functional components were defined: (1) e-views, (2) e-visits, and (3) e-requests for prescription renewal.

E-views refers to the function allowing users to view parts of their electronic health record, including reports, discharge summaries, allergies, demographics, and their ambulatory medication list. They could also view upcoming appointments and a list of people to whom the users had given consent to access their chart, and they could view and send requests to update their demographic information.

E-visits refers to the function enabling secure messaging with their primary clinician or most responsible physician.

E-requests for prescription renewal refers to the function enabling electronic prescription renewal.

Recommended frequency of use was not specified to users; rather, it was recommended to utilize educational resources, pamphlets, website, and other materials to support them with navigation within the system, as needed. Users received email notifications when new information was available in the portal.

### Portal Implementation and Enrollment

The portal implementation project was sponsored by Canada Health Infoway (CHI) and developed by Meditech to leverage the EMR’s data. Version 1 of the portal was released to the organization in December 2014. A total of 9 users pilot-tested the portal in December 2014 and provided feedback before it was rolled out to the entire organization later that month. Increased functionality and updates will be available with the next scheduled upgrade on November 1, 2016. There were no changes to the portal over the course of the data collection period (Meditech 6.07, Portal 1.0).

Enrollment was limited to inpatients or outpatients registered at Ontario Shores and their proxy users. Access and services were provided at no cost to the patient, and users continued to have access to their records following discharge.

At this stage the level of human involvement was high. At an organizational level, care providers, health care professionals, information technology, clinical informatics, health information management, and professional practice were involved in the planning and implementation of the portal. CHI provided additional support as project sponsors. They provided a detailed project structure and benefits evaluation delivery model [15] and were available as a resource and guide throughout the course of the project. Clinicians received training through a video and access to a demonstration account to experience portal use and a learning management system module. Clinicians received further training through the medical advisory council, nursing council, other professional councils, and on-unit services. Training was included in clinical orientation for new hires. A process to support clinicians with enrolling users was built into the EMR. Formal training was not provided to users, but support sessions were facilitated by peer support specialists and the engagement coordinator and available for users to attend on an ongoing basis.

### Benefits Evaluation

A standard benefits evaluation model and framework was used to evaluate this initiative [15]. CHI designed a model for completing benefits evaluations for information systems that is based on the DeLone and McLean Information Systems Success model and takes 6 interdependent variables into account: system quality, information quality, system use, user satisfaction, individual impact, and organizational impact [16]. This benefits evaluation framework focuses on the relationship between the implementation of an effective solution, the adoption of the solution, and the resulting effects. Applying this evaluation method and framework is effective in understanding progress made toward objectives, identifying barriers, and communicating successes [15]. This evaluation further utilized Infoway’s System and Use Survey Tool (SUS) from the system, information, service, use, and satisfaction indicators of the benefits evaluation framework to evaluate the implementation of this patient portal.

### Outcomes Measures

Table 1 presents the study timeline.

**Table 1:** Study timeline.

Period	Dates	Activity
Preimplementation (2014)	January 2014 to December 2014	Used to compare administrative efficiencies and productivity (retrospective analysis based on those who enrolled after going live).
Go live	December 2014	Implementation date.
Recruitment	December 2014 to December 2015	Ongoing recruitment. Completion of preportal surveys.
MHRM^a^ (>6 months) follow-up	May 2015 to December 2015	Completion of postportal survey (MHRM).
SUS^b^ (>3 months) follow-up	March 2015 to December 2015	Completion of SUS.
Postimplementation (2015)	January 2015 to December 2015	Post–portal implementation efficiencies and productivity.

^a^MHRM: Mental Health Recovery Measure.

^b^SUS: System and Use Survey Tool.

#### Demographics

Demographics (age and sex) for the overall populations were extracted from the EMR data repository via structured query language (SQL) report. Factors related to diagnosis, such as symptoms and severity, may affect portal use; however, because a number of patients had multiple diagnoses or misdiagnoses that were changed over the course of their admission, a clear description of participants’ diagnoses was not possible. Demographics for the subset of individuals completing the Web-based surveys were self-reported.

#### Portal Usage

The number of patients who registered for the portal and number of times each function was used was pulled from the data repository via SQL reports.

#### Productivity

Appointments missed by users and nonusers were pulled from EMR reporting data for the year before (2014) and the year of (2015) portal implementation.

#### Administration Efficiencies

The number of requests for information for users and nonusers was pulled from the EMR reporting data for the year before (2014) and the year of (2015) portal implementation.

#### Surveys

##### Mental Health Recovery Measure

Portal users were prompted to complete the Mental Health Recovery Measure (MHRM) at registration and 6 and 10 months following portal registration. The MHRM includes 8 recovery domains, which were examined to determine changes in recovery across the study period. Activation is seen as central to self-management, which literature indicates is linked to improving patient involvement in and having a more patient-centered organization of health care delivery [17]. Because these concepts align with the MHRM, it was chosen as a proxy measure for activation since fiscal constraints prevented the use of more traditional measures of activation. A link to the Web-based survey was available on the portal. The survey did not link to the user’s account and therefore results were anonymous. An email reminder was sent at 6 and 10 months to prompt completion of the follow-up survey.

##### System and Use Survey Tool

A link to the SUS was available on the portal 6 and 10 months after portal registration to examine users’ experiences with e-visits, e-views, and e-requests for prescription refill. A small subset of users pilot-tested the surveys at 3-month follow-up. Because no changes were made, these results were included in the analysis. Free-text answers to the SUS (administered as described above) provided qualitative feedback regarding experiences with portal use.

### Bias

The design of this study may introduce bias when comparing portal users with nonusers for organizational measures as the users were interested in and motivated to use the portal, which may translate into increased interest and motivation to participate in treatment.

### Sample Size

The entire organizational patient population was used for observation. The target sample size for survey completion was 60, based on the CHI (study sponsor) statement of work.

### Statistical Methods

Data for analyses were extracted through reporting software interfacing with the organization’s data repository and exported into Microsoft Excel 2010 for data analyses. Descriptive analyses were completed by calculating the number and percentage of service users who registered on the portal and the average of the number of log-ins per user from December 2014 to December 2015 (ie, data usage).

Missed appointment (ie, appointment kept vs appointment missed) and requests for information (ie, health information requests made vs health information not requested) data for users and nonusers were inputted to OpenEpi (version 3) [18] to calculate the odds ratio (OR) for the 2014 and 2015 data.

Changes in the overall MHRM and each of the recovery domains were examined using *t* tests [19]. Basic coding was completed for the free-text sections of the SUS. As participation in the 10 months or more follow-up surveys was low, responses to 6 and 10 months or more follow-up surveys were combined for MHRM and SUS analyses to meet CHI requirements.

## Results

### Demographics

Age and sex data were available for 3158 patients who were admitted between December 2014 and November 2015 and for 432 of the participants who registered for portal access in the same time frame. A similar proportion of patients (1756/3158, 55.6%) and portal users (266/432, 61.6%) were female. Age distribution was relatively similar, although older adults (aged ≥65 years) may have been slightly underrepresented in the subset of portal users (Table 2).

**Table 2:** Age distribution in the whole organization and in portal users.

Age range	Organization N (%)	Portal users N (%)
Total	3158 (100)	432 (100)
Under 20	577 (18.27)	60 (13.9)
20-34	887 (28.09)	169 (39.1)
35-49	632 (20.01)	123 (28.5)
50-64	561 (17.76)	71 (16.4)
65-74	197 (6.24)	6 (1.4)
75-84	176 (5.57)	2 (0.4)
Over 84	128 (4.05)	1 (0.2)

### Portal Usage

Over the year-long follow-up period, 461 service users (44% male, n=203) registered for the portal and were designated as users. The majority of users were between the ages of 25 and 34 years. The portal was used 4761 times with the majority of log-ins for e-views (n=4539, 95.3%), followed by e-visits (n=210, 4.4%) and e-renewal of prescriptions (n=12, 0.3%).

### Productivity

In 2014 (the year before the portal launch), the odds of a user attending a scheduled appointment were 17% greater than that of nonusers (OR 1.17, 95% CI 1.08-1.26). In 2015 (the year of the follow-up period), the odds of a user attending a scheduled appointment were 67% greater than that of nonusers (OR 1.67, 95% CI 1.56-1.79).

### Administrative Efficiencies

In the entire population, there was a 61% decrease in the number of requests for information from 206 in 2014 to 80 in 2015. In users, there was an 86% decrease in the number of requests for information from 23 in 2014 to 3 in 2015. In nonusers, there was a 57% decrease in the number of requests for information from 183 in 2014 to 77 in 2015.

### Surveys

In total, 91 users completed the SUS immediately following registration, and 65 users completed the SUS at combined follow-up. The median and mode response period was the 6-month follow-up.

#### Mental Health Recovery Measure

Self-reported demographics (Table 3) were similar between those completing the MHRM at registration (44% males with a median age category of 20-34 years) and follow-up (41% males with a median age category of 20-34 years). Table 4 shows the change in MHRM scores. The total MHRM score increased from 70.4 (SD 23.6; n=79) to 81.7 (SD 25.1; n=54) at follow-up (*P*=.01). Of the 8 domains, 7 increased from baseline to follow-up (Overcoming Stuckness, Self-Empowerment, Basic Functioning, Overall Well-Being, New Potentials, Spirituality, Advocacy/Enrichment; all *P*<.05).

#### System and Use Survey Tool

Of those who completed the SUS at follow-up (n=65), 48% (n=31), 22% (n=14), and 34% (n=22) reported that they utilized the e-views, e-renewal of prescriptions, and e-visits, respectively. Few users completed free-text questions of the SUS at follow-up (n=16); 3 themes each were identified for e-views and e-requests for prescription refill, and 2 themes were identified for e-visits (Table 5).

**Table 3:** Self-reported demographics of users completing the Mental Health Recovery Measure survey at portal registration and follow-up.

Demographic information		Registration (N=91) n (%)	Follow-up (N=65) n (%)
**Sex**		n=86	n=51
	Male	38 (44)	21 (41)
	Female	48 (56)	30 (59)
**Age category, years**		n=87	n=50
	Under 20	18 (21)	6 (12)
	20-34	26 (30)	18 (36)
	35-49	26 (30)	15 (30)	
	50-64	15 (17)	9 (18)
	65-74	1 (1)	1 (2)
	75-84	1 (1)	1 (2)

**Table 4:** Differences between baseline and follow-up in the 8 domains of the Mental Health Recovery Measure.

MHRM^a^ domain	Baseline		≥6-Month follow-up		Pre-post differences^b^			
	n	Mean (SD)	n	Mean (SD)	Mean difference	Degrees of Freedom (df)	*t* test	*P* value^c^	
Overcoming Stuckness	91	10.8 (3.0)	55	11.9 (2.6)	−1.0	143	−2.121	.04
Self-Empowerment	90	10.1 (3.8)	55	11.5 (4.0)	−1.3	110	−2.019	.04
Learning and Self-Redefinition	91	10.6 (3.6)	56	11.3 (3.3)	−0.6	144	−1.104	.27	
Basic Functioning	90	9.2 (3.4)	55	10.8 (3.8)	−1.6	142	−2.674	.01
Overall Well-Being	92	7.9 (4.2)	56	9.9 (4.2)	−2.1	111	−2.856	.005
New Potentials	88	9.1 (4.0)	56	10.5 (3.7)	−1.3	141	−2.052	.04
Spirituality	92	3.9 (2.5)	56	4.9 (2.5)	−1.0	145	−2.426	.02
Advocacy/Enrichment	92	9.1 (3.0)	54	10.9 (3.6)	−1.9	143	−3.404	.001
Total	79	70.5 (23.6)	54	81.7 (25.1)	−11.3	130	−2.636	.01

^a^MHRM: Mental Health Recovery Measure.

^b^Pre refers to baseline and post refers to ≥6-month follow-up.

^c^Statistical significance was defined as *P*<.05.

**Table 5:** Thematic analysis of free-text questions of the System and Use Survey.

Function	Benefits	Improvement
E-views	Autonomy: “It is an excellent tool to cultivate autonomy.” “Just having my own access has given me freedom as a patient.”	PHI^a^ not up-to-date: “The only report that was uploaded was from a psychologist that I saw a few months ago. No other reports in the past 6 months have been uploaded to the patient portal.” More information: “My file doesn’t show history of visits, but just appointment dates.”
E-requests for prescription refill	User-friendly: “Easy to use.” Helpful: “This system is very helpful for appointment reminders.” Satisfaction: “I am happy to see it works.”	
E-visits	Efficiencies: “The system saves a lot of time and money.” Satisfaction: “I’m happy with the system.”	

^a^PHI: personal health information.

## Discussion

### Principal Findings

This study is the first to report the outcomes of the implementation of an EMR-linked portal for inpatients and outpatients receiving services at a tertiary facility specializing in severe and persistent mental illness. The novel findings of this study are that implementation of the portal for inpatients and outpatients resulted in activation of service users and/or carers and in improved recovery scores according to the MHRM domains. At the organizational level, productivity was increased with fewer missed appointments and administrative efficiencies were realized with a reduced number of requests for information in the year following compared with the year before portal implementation.

### Strengths and Limitations

The main strength of this study is that data to examine organizational productivity and administrative efficiencies were available through EMR reporting software for the whole organizational population. Users self-selected registration and enrollment; therefore, the results of this study reflect actual use as we may expect this sample to be reflective of the population who would choose to use the portal in reality. Additionally, research personnel had minimal effect on the implementation of the portal. Because the entire patient population of the hospital and its associated clinics was followed up for the duration of this study, it has high internal validity. Results may be generalizable to other tertiary care mental health hospitals and outpatient clinics with similar organizational context; however, because the results are specific to this organization, generalizability to other contexts may be limited. This study is limited in that there is no control group for the MHRM. Changes in recovery over time may be a result of continuing mental health treatment and may not be associated with activation or portal use. Results may have been stronger if the well-validated Patient Activation Measure (PAM) was used to measure patient activation instead of the MHRM; however, patient activation and recovery are strongly associated [3]. Hence, it was determined that the MHRM would be an acceptable surrogate measure because budgetary constraints prevented use of the PAM. Convenience sampling was used to recruit the subset of users completing the SUS and this subset was not necessarily representative of all the users. Additionally, the administration of the surveys via anonymous Web-based survey software ensured confidentiality but prevented analysis using repeated-measures design. It is unknown how many (if any) of the users completed the survey at both baseline and follow-up or if these samples are different in composition. Demographics suggest compositions were similar.

### Comparison With Prior Work

In the literature, the effects of patient portal implementation on organizational productivity and administrative efficiencies are equivocal [7,8,11]. In this study, the odds of portal users attending an appointment were 17% greater than that of nonusers before portal implementation and 67% greater than that of nonusers in the year following portal implementation, showing increased organizational productivity. Administrative efficiencies were also realized with an overall 61% decrease in the number of requests for information with an 86% and 57% decrease in users and nonusers, respectively. Overall, the estimated administrative time efficiencies related to requests for information by users was low (10-40 hours; data not shown) because of the small number of requests made by users in both 2014 and 2015. The results, however, suggest that with increased access to information and/or activation of users, considerable improvements in time efficiencies could be realized.

One of the primary purposes of portal implementation was to activate patients and/or carers to improve outcomes and recovery. A study examining the effects of patient portals on patient activation in acute care settings showed no association between patient activation and use of the patient portal [20]. In our study, patient activation, assessed by the overall MHRM score, increased over the follow-up period suggesting that engagement with the patient portal increased activation. It should be considered, however, that the purpose of recovery-oriented mental health treatment is to help patients reach their personal goals, which requires a certain amount of activation. Patient portals may be beneficial in this clinical population as increased activation through treatment may motivate portal use and portal access may support goal achievement. Future research may be warranted to examine these relationships to enable portal functionalities to optimally support patient recovery.

The overall MHRM score and 6 of the 8 recovery domains were improved over the follow-up period. This study is the first to explore the effects of patient portal implementation on recovery in its users. The change in MHRM over the 6-10 month follow-up period (baseline, 70.7; follow-up, 81.7) was similar to the change in MHRM over a 3-6 month “Wellness Management and Recovery” program delivered to persons with mental illness (baseline, 80.2; follow-up, 88.4) [21]. Because there was no control group, it is uncertain whether improvements in recovery were accelerated by the patient portal or whether they were usual improvements with treatment. The fact, however, that this study elicited similar changes in MHRM as an intensive wellness management and recovery program suggests this is an important topic for future research.

### Conclusions

In conclusion, this benefits evaluation provides early evidence to suggest that access to electronic health records through a patient portal may have positive effects on patient activation and recovery in a population with serious and persistent mental illness. With the current functionality, there was a notable improvement in productivity with lower odds of a missed appointment for the users compared with nonusers. Future research is planned to conduct focus groups to more thoroughly examine patient experiences and to examine longitudinal effects of increased portal functionalities on mental health symptoms, recovery, and health care utilization.

## Appendix

Multimedia Appendix 1 CONSORT EHEALTH form V1.6.2 [13].

## Abbreviations

CHI: Canada Health Infoway
CONSORT-EHEALTH: Consolidated Standards of Reporting Trials of Electronic and Mobile Health Applications and Online Telehealth
EMR: electronic medical record
MHRM: Mental Health Recovery Measure
OR: odds ratio
PAM: Patient Activation Measure
SQL: structured query language
STROBE: Strengthening the Reporting of Observational Studies in Epidemiology
SUS: System and Use Survey Tool

## References

[1] Whiteford HA, Degenhardt L, Rehm J, Baxter AJ, Ferrari AJ, Erskine HE, Charlson FJ, Norman RE, Flaxman AD, Johns N, Burstein R, Murray CJL, Vos T (2013). Global burden of disease attributable to mental and substance use disorders: findings from the Global Burden of Disease Study 2010. Lancet.

[2] Shepherd GB, Boardman J, Slade M https://www.centreformentalhealth.org.uk/making-recovery-a-reality.

[3] Kukla M, Salyers MP, Lysaker PH (2013). Levels of patient activation among adults with schizophrenia: associations with hope, symptoms, medication adherence, and recovery attitudes. J Nerv Ment Dis.

[4] Hibbard JH, Stockard J, Mahoney ER, Tusler M (2004). Development of the Patient Activation Measure (PAM): conceptualizing and measuring activation in patients and consumers. Health Serv Res.

[5] Jerofke T, Weiss M, Yakusheva O (2014). Patient perceptions of patient-empowering nurse behaviours, patient activation and functional health status in postsurgical patients with life-threatening long-term illnesses. J Adv Nurs.

[6] Irizarry T, DeVito DA, Curran CR (2015). Patient portals and patient engagement: a state of the science review. J Med Internet Res.

[7] Ammenwerth E, Schnell-Inderst P, Hoerbst A (2012). The impact of electronic patient portals on patient care: a systematic review of controlled trials. J Med Internet Res.

[8] de Lusignan S, Mold F, Sheikh A, Majeed A, Wyatt JC, Quinn T, Cavill M, Gronlund TA, Franco C, Chauhan U, Blakey H, Kataria N, Barker F, Ellis B, Koczan P, Arvanitis TN, McCarthy M, Jones S, Rafi I (2014). Patients' online access to their electronic health records and linked online services: a systematic interpretative review. BMJ Open.

[9] Kruse CS, Bolton K, Freriks G (2015). The effect of patient portals on quality outcomes and its implications to meaningful use: a systematic review. J Med Internet Res.

[10] Jilka SR, Callahan R, Sevdalis N, Mayer EK, Darzi A (2015). Nothing about me without me: an interpretative review of patient accessible electronic health records. J Med Internet Res.

[11] Otte-Trojel T, de Bont A, Rundall TG, van de Klundert J (2014). How outcomes are achieved through patient portals: a realist review. J Am Med Inform Assoc.

[12] von Elm E, Altman DG, Egger M, Pocock SJ, Gøtzsche PC, Vandenbroucke JP (2007). The Strengthening the Reporting of Observational Studies in Epidemiology (STROBE) statement: guidelines for reporting observational studies. Lancet.

[13] Eysenbach G, CONSORT-EHEALTH Group (2011). CONSORT-EHEALTH: improving and standardizing evaluation reports of Web-based and mobile health interventions. J Med Internet Res.

[14] https://www.ipc.on.ca/wp-content/uploads/Resources/pbd-primer.pdf.

[15] https://www.infoway-inforoute.ca/en/component/edocman/resources/reports/benefits-evaluation/2911-methodology-for-administering-a-system-and-use-survey?Itemid=101.

[16] Delone WH, McLean ER (2003). The DeLone and McLean Model of Information Systems Success: a ten-year update. J Manage Inf Syst.

[17] Rademakers J, Jansen D, van der Hoek L, Heijmans M (2015). Clinicians' beliefs and attitudes toward patient self-management in the Netherlands; translation and testing of the American Clinician Support for Patient Activation Measure (CS-PAM). BMC Health Serv Res.

[18] Dean AG, Sullivan KM, Soe MM http://openepi.com/TwobyTwo/TwobyTwo.htm.

[19] Bullock W (2009). The Mental Health Recovery Measure (MHRM): Updated Normative Data and Psychometric Properties.

[20] Ancker JS, Osorio SN, Cheriff A, Cole CL, Silver M, Kaushal R (2014). Patient activation and use of an electronic patient portal. Inform Health Soc Care.

[21] Bullock WA, Sage J, Hupp D, Ozbey T, O'Rourke M, Smith MK (2009). From illness to wellness: an evaluation of Ohio's Wellness Management and Recovery (WMR) program in community mental health and consumer-operated service agencies. N Res Ment Health.

